# Identification and expression analysis of *OsLPR* family revealed the potential roles of *OsLPR3* and *5* in maintaining phosphate homeostasis in rice

**DOI:** 10.1186/s12870-016-0853-x

**Published:** 2016-10-03

**Authors:** Yue Cao, Hao Ai, Ajay Jain, Xueneng Wu, Liang Zhang, Wenxia Pei, Aiqun Chen, Guohua Xu, Shubin Sun

**Affiliations:** 1 State Key Laboratory of Crop Genetics and Germplasm Enhancement, Key Laboratory of Plant Nutrition and Fertilization in Low-Middle Reaches of the Yangtze River, Ministry of Agriculture, Nanjing Agricultural University, Nanjing, 210095 China; 2 National Research Centre on Plant Biotechnology, Lal Bahadur Shastri Building, Pusa Campus, New Delhi, 110012 India; 3 State Key Laboratory of Crop Genetics and Germplasm Enhancement, Key Laboratory of Plant Nutrition and Fertilization in Low-Middle Reaches of the Yangtze River, Ministry of Agriculture, College of Resources and Environmental Science, Nanjing Agricultural University, Nanjing, 210095 China

**Keywords:** Rice, Phosphate deficiency, *OsLPR* family, *OsLPR3*, *OsLPR5*, Phosphate homeostasis

## Abstract

**Background:**

Phosphorus (P), an essential macronutrient, is often limiting in soils and affects plant growth and development. In *Arabidopsis thaliana, Low Phosphate Root1* (*LPR1*) and its close paralog *LPR2* encode multicopper oxidases (MCOs). They regulate meristem responses of root system to phosphate (Pi) deficiency. However, the roles of *LPR* gene family in rice (*Oryza sativa*) in maintaining Pi homeostasis have not been elucidated as yet.

**Results:**

Here, the identification and expression analysis for the homologs of *LPR1/2* in rice were carried out. Five homologs, hereafter referred to as *OsLPR1-5*, were identified in rice, which are distributed on chromosome1 over a range of 65 kb. Phylogenetic analysis grouped *OsLPR1/3/4/5* and *OsLPR2* into two distinct sub-clades with *OsLPR3* and *5* showing close proximity. Quantitative real-time RT-PCR (qRT-PCR) analysis revealed higher expression levels of *OsLPR3-5* and *OsLPR2* in root and shoot, respectively. Deficiencies of different nutrients ie, P, nitrogen (N), potassium (K), magnesium (Mg) and iron (Fe) exerted differential and partially overlapping effects on the relative expression levels of the members of *OsLPR* family*.* Pi deficiency (−P) triggered significant increases in the relative expression levels of *OsLPR3* and *5*. Strong induction in the relative expression levels of *OsLPR3* and *5* in *osphr2* suggested their negative transcriptional regulation by *OsPHR2*. Further, the expression levels of *OsLPR3* and *5* were either attenuated in *ossiz1* and *ospho2* or augmented in rice overexpressing *OsSPX1*.

**Conclusions:**

The results from this study provided insights into the evolutionary expansion and a likely functional divergence of *OsLPR* family with potential roles of *OsLPR3* and *5* in the maintenance of Pi homeostasis in rice.

**Electronic supplementary material:**

The online version of this article (doi:10.1186/s12870-016-0853-x) contains supplementary material, which is available to authorized users.

## Background

Phosphorus (P), one of the essential macronutrients, is required for several biochemical and physiological processes and is a component of key macromolecules including nucleic acids, ATP and membrane phospholipids [1]. P is absorbed from rhizosphere as phosphate (Pi), which is often not easily available to plants due to its slow diffusion rates in soils and/or fixation as immobile organic Pi [2]. Limited Pi availability adversely affects growth and development of plants [3].

In *Arabidopsis thaliana,* Pi deficiency triggers progressive loss of meristematic activity in primary root tip thereby inhibiting primary root growth (PRG) [4]. *LPR1* (At1g23010) and its close paralog *LPR2* (At1g71040), encoding multicopper oxidases (MCOs), are major quantitative trait loci (QTLs) associated with Pi deficiency-mediated inhibition of PRG [5, 6]. Loss-of-function mutations in *LPR1* and *LPR2* affect Pi deficiency-mediated inhibition of PRG [6]. However, unlike Arabidopsis, Pi deficiency either does not exert any significant effect on PRG of taxonomically diverse dicots and monocots [7, 8] or triggeres augmented PRG in rice [9, 10]. These studies suggested that Pi deficiency-mediated inhibition of PRG is not a global response across different plant species. This raised an obvious question about the likely role of homologs of *LPR1/2* particularly in species such as rice in which Pi deficiency has a rather contrasting influence on PRG.

Nuclear-localized *SIZ1* (At5g60410) encodes a small ubiquitin-like modifier (SUMO) E3 ligase1 and sumoylates transcription factor (TF) *PHR1* (At4g28610) in Arabidopsis [11]. *PHR1* plays a pivotal role in regulating the expression of Pi 3starvation-responsive (PSR) genes whose promoters are enriched with PHR1-binding sequence (P1BS) motif [12]. *PHR1* is a pivotal upstream component of the Pi sensing and signaling cascade comprising *miR399*s, *IPS1* (At3g09922), *PHO2* (At2g33770), *SPX1* (At5g20150), Pi transporters *Pht1;8* (At1g20860),*Pht1;9* (At1g76430) and a subset of other PSR genes [13–15]. Interestingly though, promoters of both *LPR1* and *LPR2* do not have P1BS motif, which suggests a lack of any regulatory influence of *PHR1* on the expression of these genes. Therefore, the identification of TFs that regulate *LPR1/2* solicits further studies.

Rice, one of the most important cereal crops, feeds over one-third population of the world and sometimes is the only source of calories [16, 17]. Rice is often cultivated in rain-fed system on soils that are poor in Pi availability, which affects its growth and development and consequently the yield potential [16]. Therefore, it is increasingly becoming imperative to decipher the intricacies involved in the maintenance of Pi homeostasis for developing rice with higher Pi use efficiency for the sustainability of agriculture. Pi starvation signal transduction pathway is highly conserved between Arabidopsis and rice [17]. In this context, several homologs of Arabidopsis in rice ie, *OsPHR2* [18, 19], *OsPHO2* [20, 21], *OsSPX1* and *OsSPX2* [22] have been functionally characterized and are pivotal components of Pi sensing and signaling cascade [17]. However, the roles of homologs of *LPR1/*2 in rice during the maintenance of Pi homeostasis have not been elucidated as yet.

In this study, the identification and expression analysis of *OsLPR1-5* in rice were carried out. Phylogenetic analysis revealed their grouping into two distinct subclades. Differential expression of these genes under both Pi-replete and Pi-deprived conditions and also under other nutrient deficiencies suggested functional divergence across them. Further, analyses of the relative expression levels of *OsLPR3* and *OsLPR5* in loss-of-function mutants (*ossiz1*, *osphr2* and *ospho2*) and transgenic rice overexpressing either *OsPHR2* or *OsSPX1* provided an insight into their potential roles in Pi sensing and signaling cascade.

## Results and discussion

### Comparative structure analysis of *LPRs* in Arabidopsis and rice

**Fig. 1:**
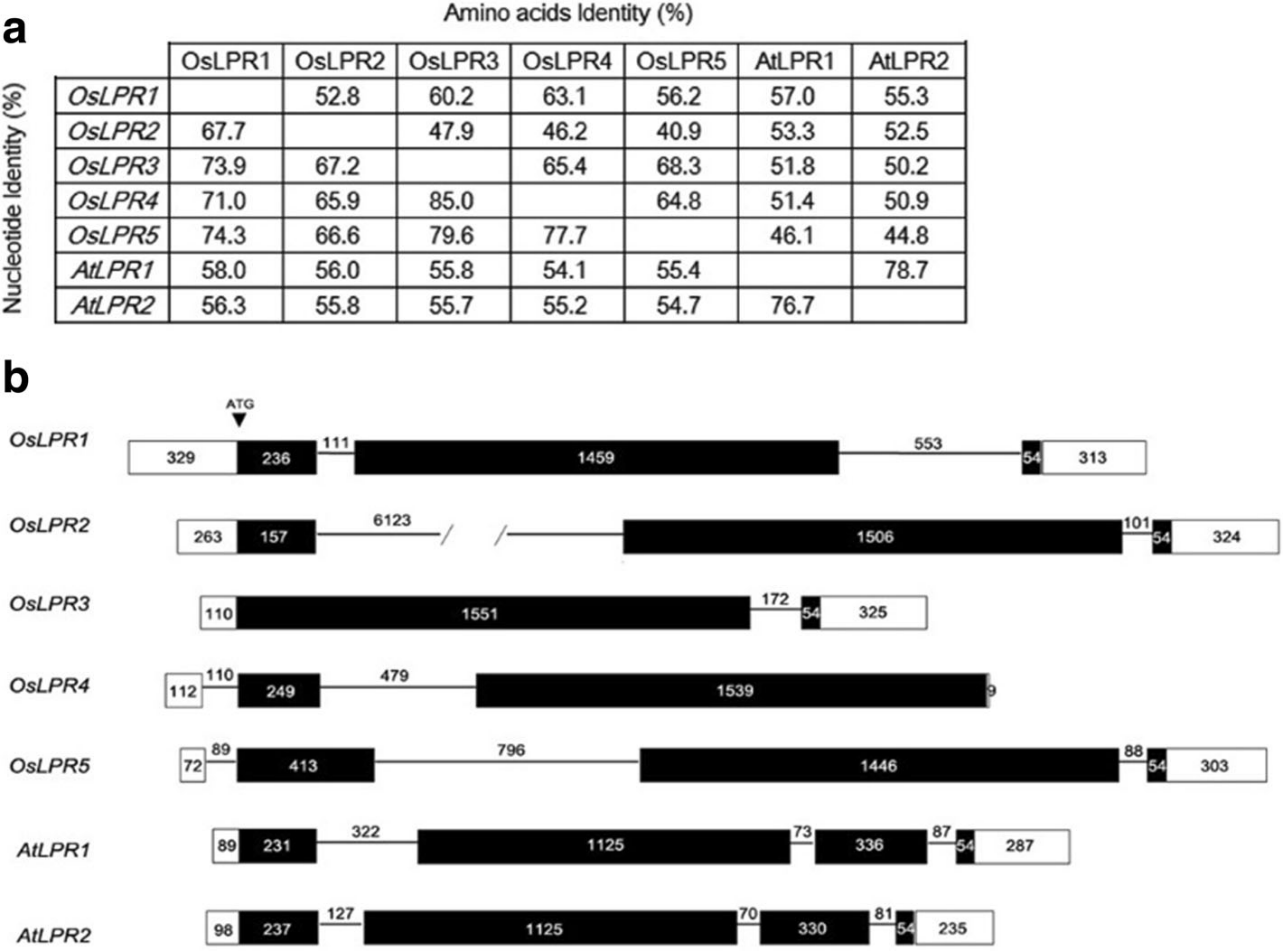
Comparative identity matrices and gene structures of *LPR* genes in Arabidopsis and rice. **a** DNAMAN 7.0 program was used for multi-sequence alignments of nucleotides and amino acids for determining per cent identity matrices across them. **b** Schematic representation of genes showing UTR (empty boxes), CDS (black boxes) and introns (black lines) with numbers indicating length of each of them

Protein sequences of Arabidopsis LPR1-2 were used as queries by TBLASTN search in National Center for Biotechnology Information (NCBI) database, which identified five homologous genes in the rice genome and hereafter referred to as *OsLPR1-5*. Details of their locus ID, cDNA accession number and protein characteristics are listed in Additional file 1. *OsLPR1-5* are localized closely within a range of 65 kb on the short arm of chromosome 1 (Additional file 2). DNAMAN 7.0 program was used for multi-sequence alignments of nucleotides and amino acids of *LPR1-2* and *OsLPR1-5* and per cent identity matrices across them were determined (Fig. 1a). Nucleotide equence identity (SI) was 85 % between *OsLPR3* and *OsLPR4* and 67.2 % between *OsLPR2* and *OsLPR3.* Amino acid SI was 68.3 % between OsLPR3 and OsLPR5 and 40.9 % between OsLPR2 and OsLPR5. The analysis suggested a relative closeness of OsLPR5 to OsLPR3 and distant from OsLPR2. Nucleotide SI of *LPR1* with *OsLPR1* and *OsLPR4* were 58 and 54.1 %, respectively. Amino acid SI varied from 57 % between OsLPR1 and LPR1 to 44.8 % between OsLPR5 and LPR2. This suggested that members of the OsLPR family are phylogenetically more closely related to each other than to LPRs. For comparative analysis of the number and position of exons and introns in *LPRs* from rice and Arabidopsis, their full-length cDNA sequences were aligned with their corresponding genomic DNA sequences (Fig. 1b). Number of exons ranged from four (*LPR1*-*2*), three (*OsLPR1*/*2*/*5*) to two (*OsLPR3/4*)*.* In rice, the longest exon varied from 1446 bp in *OsLPR5* to 1551 bp in *OsLPR3*, while it was 1125 bp in both *LPR1-2.* With a notable exception of *OsLPR4*, the last exon of *LPRs* and *OsLPRs* was 54 bp in length. Introns also exhibited variation in their number ranging from three (*LPR1*-*2* and *OsLPR5*), two (*OsLPR1*/*2*/*4*) to one (*OsLPR3*) with length varying from 70 bp in *LPR2* to 6123 bp in *OsLPR2.* Further, the 5' untranslated regions (UTR) of *OsLPR4/5* were disrupted by an intron. The analysis thus revealed both the divergence and conservation of *LPR* genes in Arabidopsi*s* and rice.

### Phylogenetic analysis of *LPR* genes

**Fig. 2:**
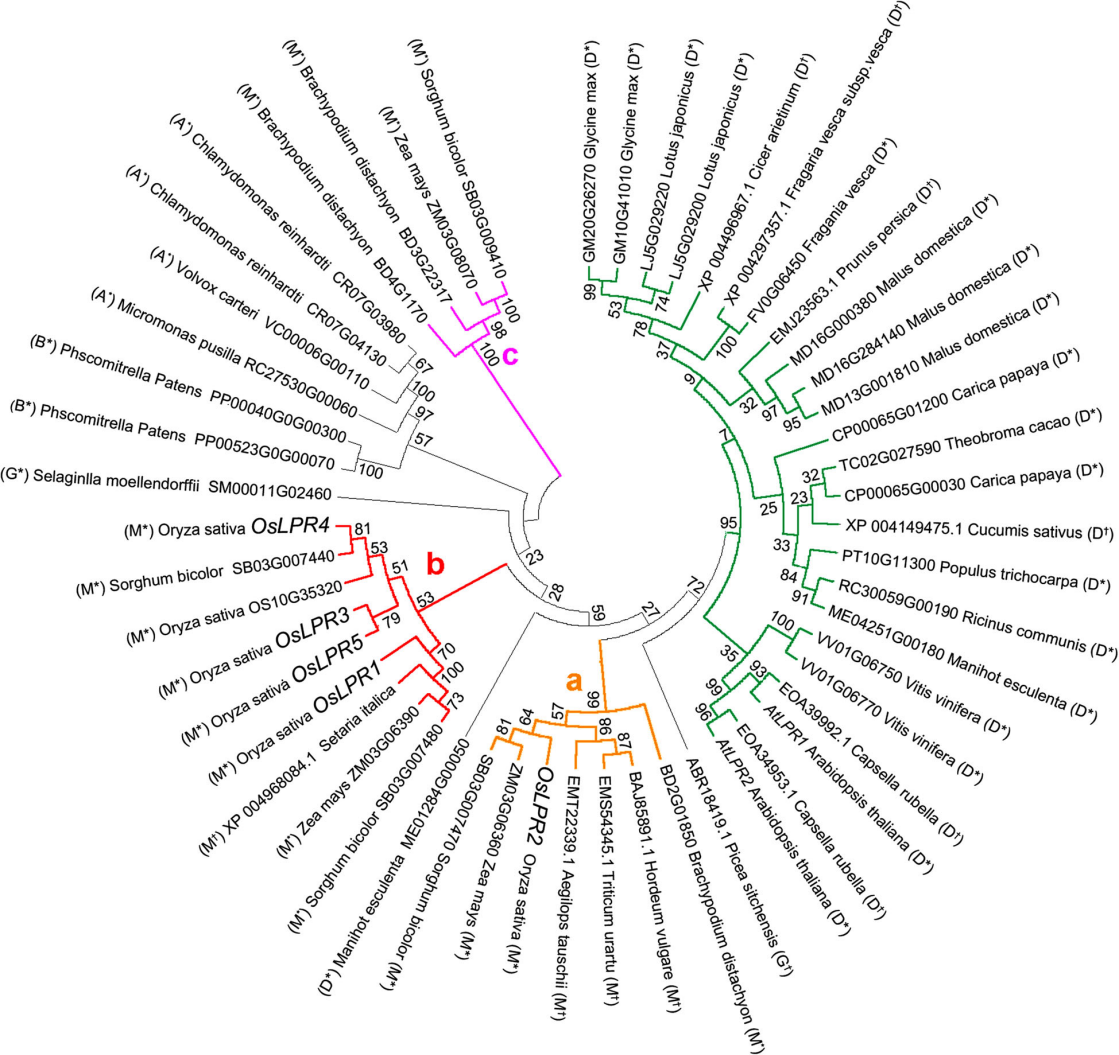
Phylogenetic analysis of *LPR* gene family in plants. Joint unrooted phylogenetic tree of 53 putative *LPR* genes from 29 different higher and lower plant species representing dicots (*D*), monocots (*M*), gymnosperms (*G*), bryophytes (*B*) and algae (*A*). * and † represent species that have been sequenced and not sequenced as yet, respectively

LPR1 and LPR2 were used as queries in the BLASTP search on NCBI and PLAZA databases, which identified 53 LPR homologs from taxonomically diverse higher (15 dicots, 8 monocots and 2 gymnosperms) and lower plants (1 bryophyte and 3 algae). An unrooted phylogenetic tree of all the homologs identified was reconstructed using MEGA 4.0 using the neighbor-joining method (Fig. 2). Monocot LPR proteins grouped into clades *a, b* and *c* represented by yellow, red and purple lines, respectively, on the phylogenetic tree. Except OsLPR2, OsLPR1 and OsLPR3-5 clubbed together in clade *b* with a closer evolutionary distance along with LPRs from *Sorghum bicolor* (SB03G007440, SB03G007480), *Setaria italic* (XP004968084.1) and *Zea mays* (ZM03G06390). Grouping of OsLPR3 and OsLPR5 in a distinct single sub-branch was consistent with their high nucleotide and amino acid SI (Fig. 1a). In a single subclade, OsLPR3 and OsLPR5 were inparalogs but outparalogs of OsLPR1/2/4. *OsLPR2* was placed in clade *a* along with LPRs from the members of the grass family ie, *S. bicolor* (SB03G007470), *Z. mays* (ZM03G06360), *Aegilop stauschii* (EMT22339.1), *Triticum urartu* (EMS54345.1), *Hordeum vulgare* (BAJ85891.1) and *Brachypodium distachyon* (BD2G01850). Orthologs of OsLPR1/2/4 were also found in other monocot species. The clade *c* comprising LPRs from *B. distachyon* (BD4G11770, BD3G22317), *Z. mays* (ZM03G8070) and *S. bicolor* (SB03G009410) revealed long evolutionary distance from both clades *a* and *b.* Although all the LPRs from dicots formed a distinct clade (green), notable exception was the placement of LPR from *Manihot esculenta* (ME01284G00050) (grey clade) between clades *a* and *b*. Both AtLPR1 and AtLPR2 exhibited close phylogenetic relationships with LPRs from *Capsella rubella* (EOA34953.1 and EOA39992.1). LPRs from gymnosperm (*Selaginella moellendorffii*), bryophyte (*Physcomitrella patens*) and algae (*Micromonas pusilla*, *Volvox carteri* and *Chlamydomonas reinhardtii*) grouped in grey clade. It is apparent from this phylogenetic analysis that LPRs in monocotyledonous species are closely related suggesting a likely duplication event preceding the split between monocots and dicots. On the contrary, LPR paralogs in dicotyledonous species were closely related indicating duplication following the split between monocots and dicots. Therefore, it could be assumed that OsLPRs may have functions similar to orthologs from other monocotyledonous species but different from LPRs in dicotyledonous species including Arabidopsis. Overall, the analysis revealed the conservation of LPRs across taxonomically diverse higher and lower plant species.

### Cu-oxidase domain analysis of LPR proteins in rice

**Fig. 3:**
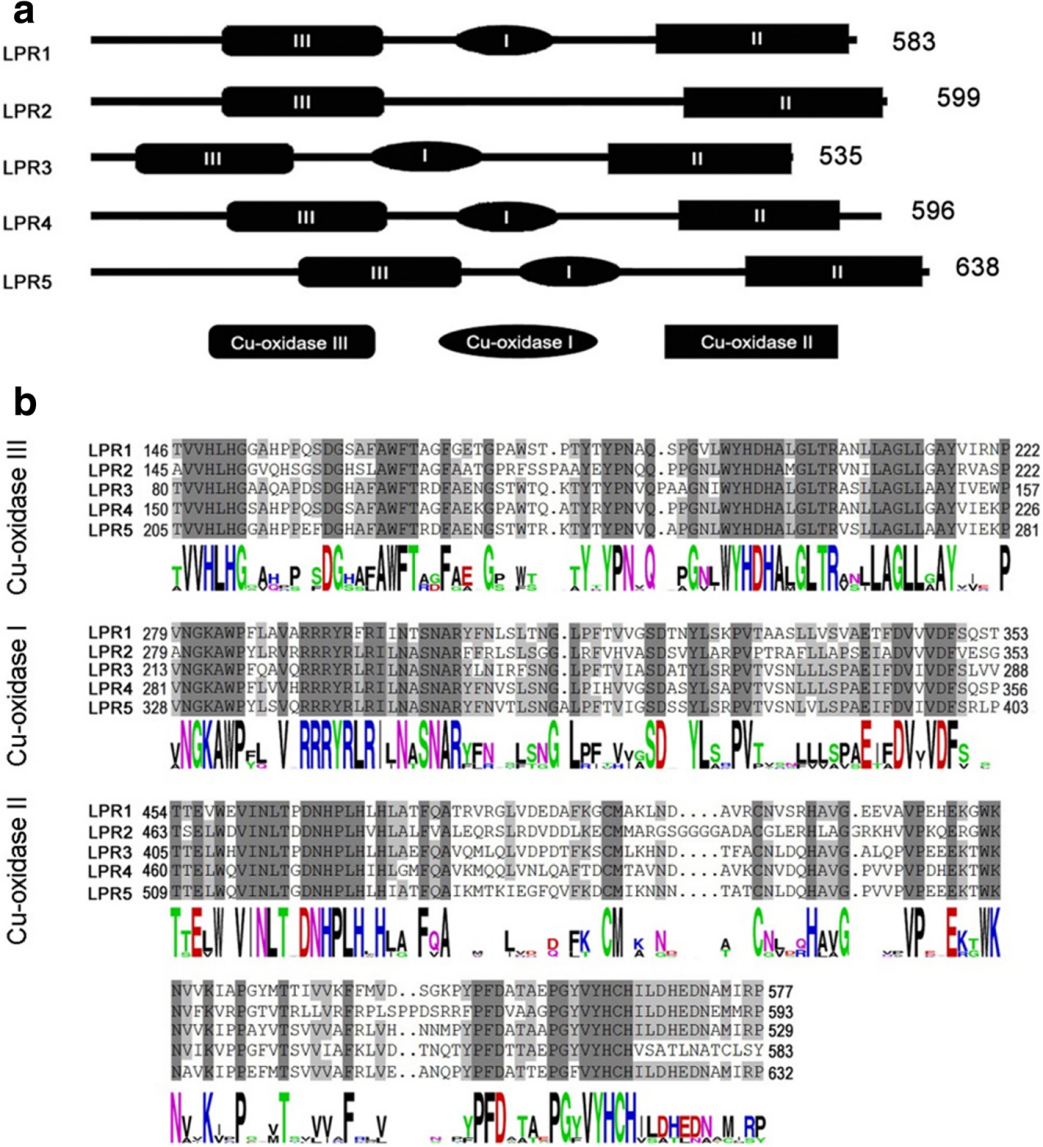
Analysis of Cu-oxidase domain structure of LPR proteins in rice. **a** Cu-oxidase I, II and III domains in OsLPR proteins are indicated by elliptic, rectangle and rounded rectangle, respectively. Number indicates length of OsLPR protein. **b** Alignment of amino acid sequences of Cu-oxidase I, II and III domains of LPR proteins in rice. Identical and similar amino acids across LPR proteins are highlighted with dark and light grey backgrounds, respectively. Consensus sequences determined by Weblogo (http://weblogo.berkeley.edu/) are presented at the bottom

Multicopper oxidase (MCO) facilitates oxidation of organic or metal ions, and trinuclear Cu cluster (TNC) is involved in the reduction of O_2_ [23]. In Arabidopsis, MCO activity of LPR proteins is pivotal for eliciting inhibition of primary root growth during Pi deficiency [6]. Pfam and NCBI protein databases (http://pfam.xfam.org/ and http://www.ncbi.nlm.nih.gov/guide/proteins/#databases) were employed for the analysis of the domain structures of Cu-oxidase 1–3 and peroxidase in LPR proteins of higher and lower plant species that have been sequenced (Additional file 3). The analysis revealed significant differences in sizes and positions of Cu-oxidase 1–3 and peroxidase domains of putative LPR proteins of *B. distachyon* (BD4G11770, BD3G22317), *Z. mays* (ZM03G8070) and *S. bicolor* (SB03G009410) compared with other LPR proteins. Further, Cu-oxidase domains were analyzed in OsLPR proteins (Fig. 3a). Cu-oxidase domains I, II and III were detected in OsLPR1, 3, 4 and 5 with a notable absence of Cu-oxidase I domain in OsLPR2. Full-length deduced polypeptides of LPR proteins comprised 535–638 amino acids. Clustal X and DNAMAN 7.0 programs were used for multiple-sequence alignment of amino acids of Cu-oxidase I, II and III domains of OsLPR proteins (Fig. 3b). The number of amino acids in Cu-oxidase I, II and III across OsLPRs were 74–75, 77–78 and 123–130, respectively. The analysis revealed significant conservation across all three domains of Cu-oxidase in OsLPRs, which is critical for the maintenance of their optimal efficacy. Michigan State University (MSU) rice database (rice.plantbiology.msu.edu/index.shtml) search resulted in the identification of another 42 genes (27 laccases, 4 L-ascorbate oxidases and 10 monocopper oxidases), which are represented by three Cu-oxidase domains. MEGA 4.0 was used for reconstructing an un-rooted dendrogram revealing phylogenetic relationship across these genes (Additional file 4). The analysis revealed a relative closeness of OsLPRs to the members of mono-copper oxidase subfamily. On the contrary, N-terminal regions of OsLPR proteins in Arabidopsis and rice showed a rather low per cent identity (Additional file 5).

### Tissue-specific expression profiles of *OsLPRs*

**Fig. 4:**
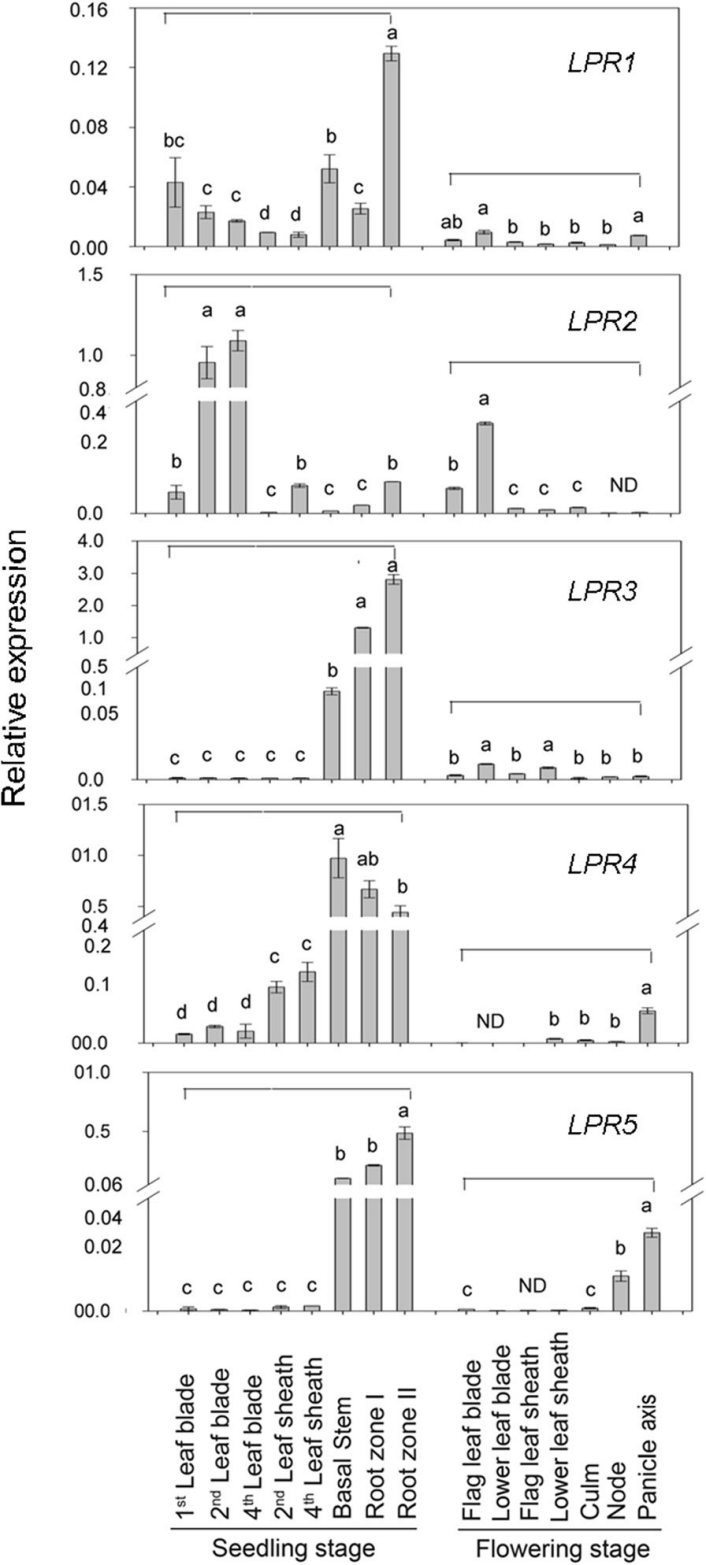
Differential tissue-specific expression of *OsLPRs*. Tissues were collected at seedling (14-d-old) and flowering (60-d-old) stages. At seedling stage, leaf blades were named as 1st to 4th from top to base. Root zones I and II represented 1 cm and >1 cm from root tip, respectively. Sheath related to each blade were numbered 2nd to 5th with 1st leaf blade being wrapped in 2nd leaf. During flowing stage, 3rd blade from top to base represented lower blade. qRT-PCR was used for determining the relative expression levels of *OsLPRs. Actin* (*OsRac1*; accession no. AB047313) was used as an internal control. Values are means ± SE (*n* = 3) and different letters indicate that the values differ significantly (*P* < 0.05)

To determine the spatiotemporal expression pattern of *OsLPRs*, qRT-PCR was performed at seedling (14-d-old) and flowering (60-d-old) stages (Fig. 4). At seedling stage, different tissues (1st, 2nd and 4th leaf blade, 2nd and 4th leaf sheath, basal stem and root zone I and II) were examined. Although expression of *OsLPR1* was detected in all the tissues of the seedlings examined, its level was significantly higher in root zone II compared with other tissues. On the contrary, expression levels of *OsLPR3* and *OsLPR5* were largely detected in root zones and basal stem with relatively low or barely detectable expression levels in leaf blades and leaf sheaths. Expression of *OsLPR4* was also relatively higher in basal stem and root zones compared with leaf sheath and leaf blade. High expression levels of *OsLPR1/3/4/5* in roots suggested their potential roles in acquisition of nutrients by roots from the rhizosphere. The expression of *OsLPR2* was significantly higher in 2nd and 4th leaf blades, moderate in 1st leaf blade, 4th leaf sheath and root zone II, and low in 2nd leaf sheath, basal stem and root zone I. This suggested a likely role of *OsLPR2* in mobilization of nutrients to shoot. At flowering stage, the expression pattern of *OsLPRs* was examined in flag leaf blade, lower leaf blade, flag leaf sheath, lower leaf sheath, culm, node and panicle axis. Although low expression of *OsLPR1* was detected in lower leaf blade and panicle axis, it could barely be observed in other tissues. *OsLPR2* showed high transcript levels in flag and lower leaf blade, low transcript levels in leaf sheath (flag and lower) and culm and was not detected in node and panicle axis. The expression of *OsLPR3* was relatively higher in lower leaf blade and lower leaf sheath compared with other tissues, while that of *OsLPR4* was significantly higher in panicle axis compared with lower leaf sheath, culm and node and remained undetected in flag leaf blade, lower leaf blade and flag leaf sheath. In the case of *OsLPR5*, the expression pattern revealed a trend similar to *OsLPR4* with a significantly higher level in panicle axis compared with other tissues. *Pht1;1* (*OsPT1*), one of the 13 *Pht1* Pi transporters in rice, expressed abundantly and constitutively in various cell types of both roots and shoots (Sun et al., 2012). Therefore, *OsPT1* was used as a positive control for determining the relative expression levels of all the members of *OsLPR* family in different tissues of 21-d-old rice seedling (Additional file 6). Overall, the relative expression levels of different members of *OsLPR* family were higher at the seedling stage compared with flowering stage. The results suggested potentially different roles of the members of *OsLPRs* in a tissue- and development-specific manner. Functional divergence is also prevalent across the members of *OsPTs* (Pi transporters) and *OsSPXs* (SPX domain-containing proteins) gene families in rice [17].

### Nutrient deficiencies affect the expression profiles of *OsLPRs*

**Fig. 5:**
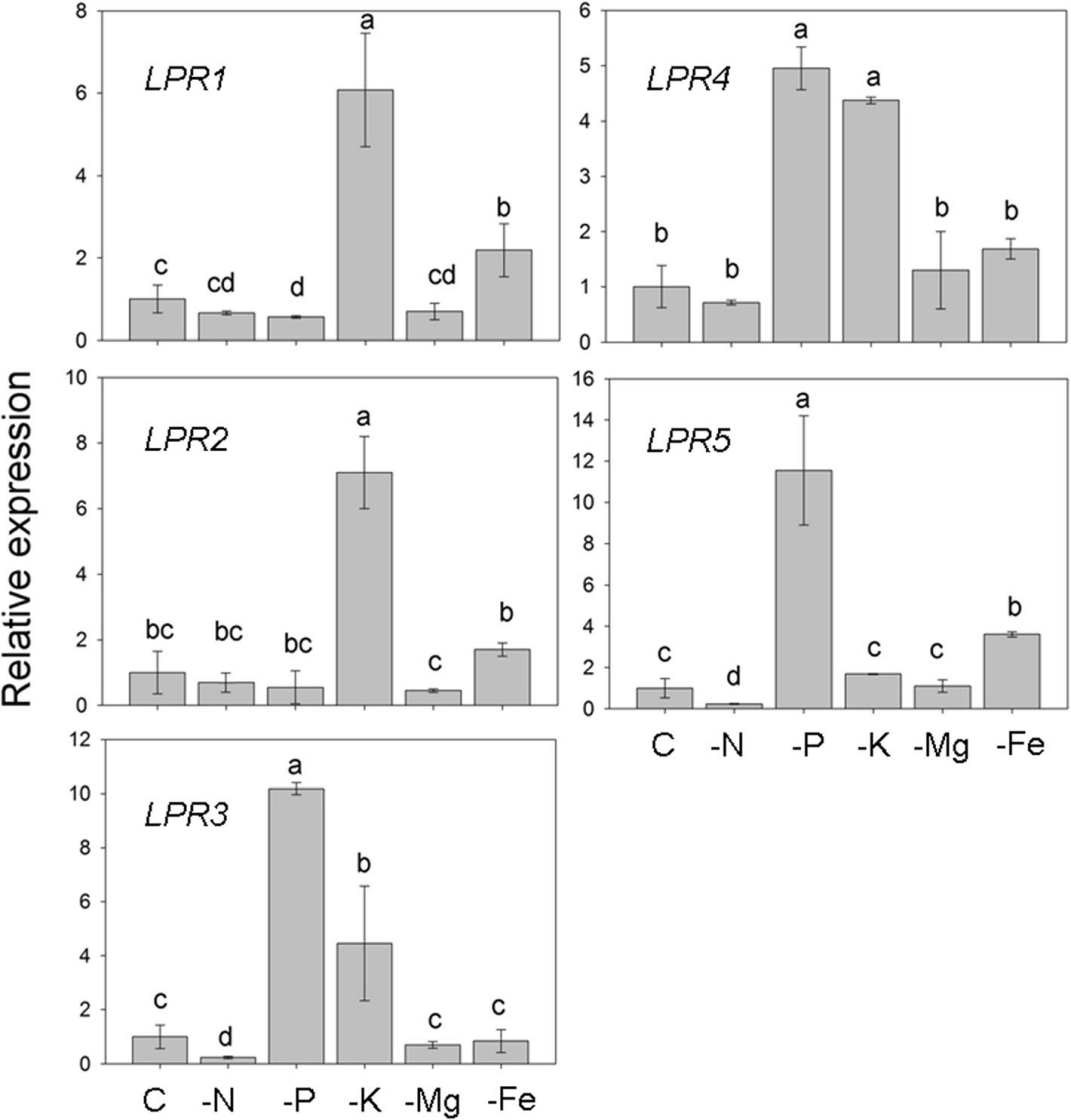
Different nutrient deficiencies exert variable effects on the expression of *OsLPRs* in roots. Rice seedlings (14-d-old) were grown in complete nutrient solution (C) and in nutrient solution deprived of one of the nutrients ie, Pi, N, K, Mg or Fe for 7 d. qRT-PCR was used for determinin g the relative expression levels of *OsLPRs* in roots. *Actin* was used as an internal control. Values are means ± SE (*n* = 3) and different letters indicate that the values differ significantly (*P* < 0.05)

Rice seedlings (14-d-old) were grown for 7 d in complete nutrient solution (C) and in nutrient solution deprived of one of the nutrients ie, Pi, nitrogen (N), potassium (K), magnesium (Mg) and iron (Fe). Roots of these seedlings were assayed for the relative expression levels of *OsLPRs* by qRT-PCR (Fig. 5). Compared with C, relative expression levels of *OsLPR1* were significantly induced under –K and –Fe conditions, attenuated under –P condition and remained unaffected under –N and –Mg conditions. Although –K triggered a significant increase in the relative expression level of *OsLPR2,* other nutrient deficiencies did not exert any significant influence on its expression level compared with C. Relative expression levels of *OsLPR3* were significantly induced under –P and –K conditions, reduced under –N condition and was unaffected under –Mg and –Fe conditions compared with C. Relative expression levels of *OsLPR4* were elevated under –P and –K conditions but other nutrient deficiencies did not exert any significant influence on its expression level compared with C. Relative expression levels of *OsLPR5* increased under –P and –Fe conditions, decreased under –N condition and remained comparable with C under –K and –Mg conditions. The analysis revealed variable effects of different nutrient deficiencies on the expression levels of *OsLPRs.* Among different nutrient deficiencies, Pi deficiency revealed wide spectrum effects ranging from induction (*OsLPR3-5*), attenuation (*OsLPR1*) and no influence (*OsLPR2*) on the relative expression level of these genes. This suggested their potentially variable and specific roles in regulating Pi homeostasis in rice. In Arabidopsis, *LPR1* has been shown to play a pivotal role in inhibition of primary root growth in response to sensing local Pi deprivation [6, 24]. However, unlike taproot system in Arabidopsis, rice has a fibrous root system [25] and deficiency of Pi triggers its elongation [9, 10, 26]. This raised a pertinent question about a likely role, if any, of any of the Pi-responsive members of *OsLPRs* in Pi deficiency-mediated developmental responses of rice root system. Analysis of their loss-of-function mutants could provide a better insight, which requires further comprehensive studies. Variable responses to Pi deficiency have also been reported for members of gene family with SPX (SYG1/PHO81/XPR1) domain, which are designated as *OsSPX1-6.* Among these, *OsSPX 1,2,3,5* and *6* are responsive to Pi starvation [27]. Although *OsSPX4* is not responsive to Pi deficiency, SPX4 interacts with OsPHR2 and negatively regulates Pi signaling and homeostasis [28]. In this context, non-responsiveness of *OsLPR2* to Pi deficiency may not completely rule out its role in Pi sensing and signaling cascade. Increase in the relative expression levels of *OsLPR3* and *OsLPR4* under both –P and –K conditions suggested cross talk between these two nutrients. A similar cross talk between P and K was also observed in soybean in which several members of *GmPT*s, a *Pht1* gene family encoding Pi transporters, were upregulated by both P and K deficiencies [29]. In another study, a high-density array comprising 1,280 genes from tomato roots revealed coordinated and coregulation of genes encoding transporters of Pi and K when deprived of either Pi or K [30]. Furthermore, microarray analysis of the global Pi deficiency response in Arabidopsis revealed significant induction in the expression levels of several genes (*KUP10, KUP11, HAK5, KAT1* and *KEA2*) encoding different K transporters [31]. Suppression and induction in the relative expression of *OsLPR1* under –P and –Fe conditions, respectively suggested their antagonistic effects on this gene. The result was consistent with an earlier study, which showed that availability of Pi exerted significant influence on the regulation of Fe-responsive genes in rice [26]. Further, availability of Fe also affects Pi deficiency-mediated morphophysiological and molecular responses in Arabidopsis [31–33]. These studies thus provided evidences of a cross talk between Pi and Fe in both rice and Arabidopsis. On the contrary, −N either exerted attenuating (*OsLPR3* and *OsLPR5*) or no effect (*OsLPR1, OsLPR2* and *OsLPR4*) on the relative expression levels of different members of *OsLPRs*. Increases in the relative expression levels of *OsLPR3* and *OsLPR5* under –P condition and their suppression under –N condition suggested an incidence of an antagonistic cross talk between these two nutrients in rice. A similar antagonistic cross talk between these two nutrients was evident in rice for a gene encoding sulfate transporter 1.2 (LOC_Os03g09970), which was up- and down-regulated in response to –P and –N conditions, respectively [34]. There are also growing evidences toward the interactions between P and N signaling pathways in Arabidopsis [35–37]. Overall, the study revealed the cross talk across different nutrients, which exerts regulatory influence on *OsLPR* family members. It is consistent with well established dogma that deficiency of one nutrient can cause imbalance of other nutrients and thereby their related morphophysiological and molecular responses [38]. On the contrary, expression levels of all the members of *OsLPRs* were not affected during Mg deficiency.

### Phosphite represses *OsLPR3/5* responses to Pi deficiency in rice

**Fig. 6:**
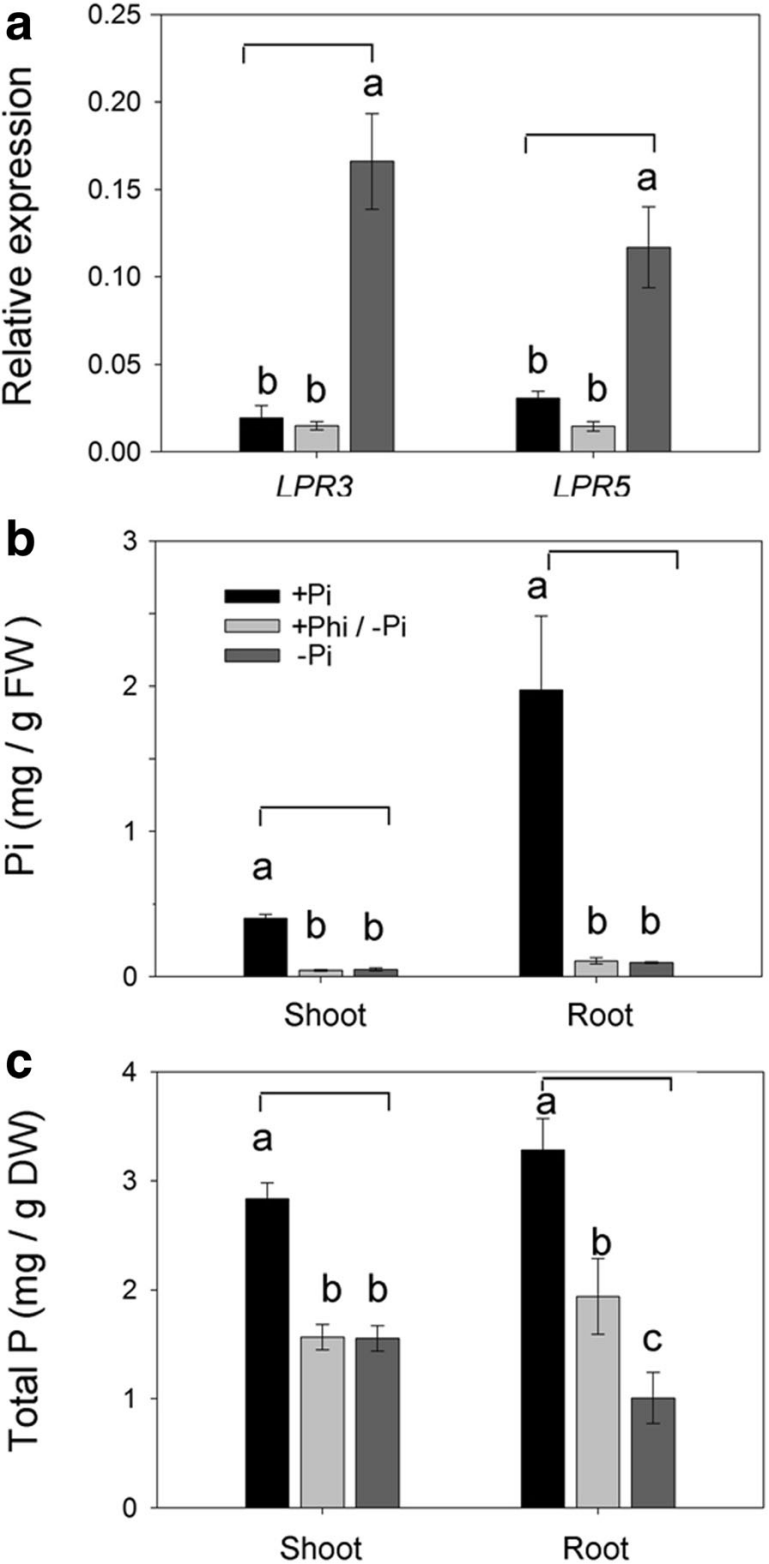
Phosphite represses the responses of *OsLPR3/5* to Pi deficiency. Rice seedlings (14-d-old) were grown under + Pi (300 μM Pi), −Pi (0 μM Pi) and + Phi/–Pi (300 μM Phi/ 0 μM Pi) conditions for 3 d. **a** qRT-PCR was used for determining the relative expression levels of *OsLPR3/5* in the roots. *Actin* was used as an internal control. Data are presented for **b** Pi content and **c** Total P and values are means ± SE (*n* = 3) with different letters indicating values that differ significantly (*P* < 0.05)

Phosphite (Phi) is a non-metabolizable analog of Pi. Phi is taken up by plant through Pi transporters, mimics Pi to some extent, interferes with Pi signaling and have been shown to suppress the coordinated expression of PSR genes in Arabidopsis [39–41]. Phi is thus a potent tool for determining whether a gene is a component of a sensing and signaling network that governs Pi homeostasis. Therefore, to compare the effects of Phi and Pi deficiency treatments on the relative expression levels of *OsLPR3/5* in roots, rice seedlings (14-d-old) were grown under + Pi (300 μM Pi), −Pi (0 μM Pi) and + Phi/–Pi (300 μM Phi/ 0 μM Pi) conditions for 3 d (Fig. 6a). There were significant increases in the relative expression levels of both *OsLPR3/5* in roots of –Pi seedlings compared with + Pi seedlings. However, the relative expression levels of these genes in + Phi/–Pi roots were significantly attenuated and became almost comparable with + P seedlings. The results provided evidence towards the involvement of *OsLPR3/5* in Pi deficiency-mediated signal transduction. The results were consistent with an earlier study reporting attenuation in the expression of Pi starvation-induced *OsIPS1* and *OsIPS2* in rice upon long-term exposure to Phi [42]. As anticipated, there were significant reductions in the contents of Pi and total P in root and shoot of –Pi seedlings compared with + Pi seedlings (Fig. 6b, c). Significant reductions in the contents of Pi (shoot and root) and total P (shoot) were also observed in + Phi/–Pi seedlings and the values were comparable with –Pi seedlings. This suggested that + Phi/–Pi and –Pi treatments treatment exerted similar attenuating influence on Pi content and total P. Notably though, total P content in + Phi/–Pi roots was significantly lower and higher compared with + Pi and –Pi roots, respectively. The results thus suggested partial influence of Phi on sensing and signaling cascade governing Pi homeostasis.

### Short- and long-term effects of Pi deficiency on the expression profiles of *OsLPRs* in the roots

**Fig. 7:**
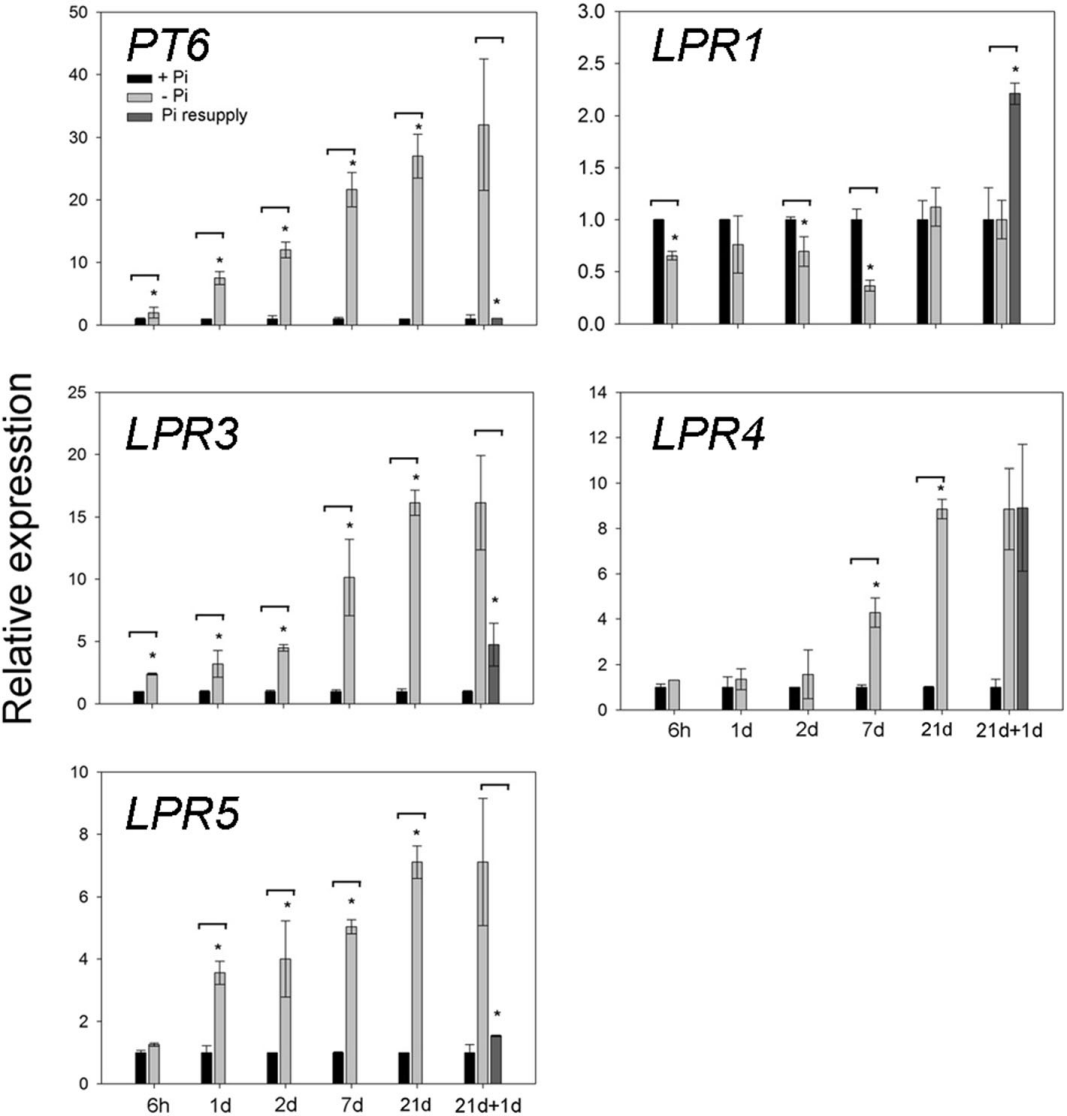
Short-and long-term effects of Pi deprivation on the expression of *OsLPRs* in roots. Rice seedlings (14-d-old) were grown under + P (300 μM Pi) and -P (0 μM Pi) conditions for 6 h, 1 d, 2 d, 7 d and 21 d. After 21 d of treatment, half of -P plants were replenished with + P for 1 d. qRT-PCR was used for determining the relative expression levels of *OsLPRs* (1, 3, 4 and 5) in root samples. Effects of Pi deprivation on their relative expression levels were also compared with Pi deficiency-induced high affinity Pi transporter *OsPT6. Actin* was used as an internal control. Values are means ± SE (*n* = 3) and asterisk indicates that the values for -P differ significantly (*P* < 0.05) compared with + P

Rice seedlings (14-d-old) were subjected to + Pi and –Pi conditions for different time intervals (6 h, 1 d, 2 d, 7 d and 21 d) and subsequently replenished with + Pi (1 d) after –Pi (21 d) treatment. An earlier study had reported complete Pi starvation of rice seedlings after 21 d of –Pi treatment [43]. High affinity Pi transporter *OsPT6* is induced rapidly and sustains induction in both roots and shoots during –Pi treatment [44]. Therefore, *OsPT6* is a potent gene for validating the fidelity of the growth conditions used for growing rice seedlings under + Pi and –Pi conditions. qRT-PCR was employed for determining the relative expression levels of *OsLPRs* (*1*, *3*, *4* and *5*) and *OsPT6* in the roots of seedlings grown under + Pi and –Pi conditions for different time intervals and upon replenishment with + Pi (Fig. 7). Relative expression levels of *OsPT6* induced rapidly during short-term (6 h) –Pi treatment, augmented commensurately during longer durations of this treatment and attenuated rapidly upon replenishment of –Pi (21 d) seedlings with + Pi (1 d). The results provided evidence towards the efficacy of the growth condition being employed in the present study for determining the temporal effects of –Pi condition on the relative expression profiles of the members of *OsLPRs.* Compared with + Pi, the relative expression levels of *OsLPR1* were significantly attenuated during –Pi treatments for 6 h, 2 d and 7 d and induced significantly upon replenishment with + Pi. On the contrary, there was a significant increase in the relative expression level of *OsLPR3* during short-term (6 h) –Pi treatment and its relative expression levels increased concomitantly with an increase in the duration of this treatment compared with + Pi. Although relative expression level of *OsLPR5* after short-term (6 h) –Pi treatment was comparable with + Pi, its levels increased significantly during prolonged (1d, 2 d, 7 d and 21 d) –Pi treatments exhibiting a trend similar to *OsLPR3.* Many of the PSR genes are known to be induced transiently during short-term –Pi treatment [45]. On the contrary, inductions in the relative expression levels of *OsLPR3* and *5* during short-term (6 h) –Pi treatment were not transient. In a global microarray analysis of spatiotemporal –Pi responses of Arabidopsis, several PSR genes involved in Pi acquisition (*Pht1;4*; [46]), mobilization (*RNS1*; [47]), phospholipid substitution (*SQD2*; [48]) and root development (*PLDZ2*; [49]) also showed a similar pattern of early and sustained induction. There were significant reductions in the relative expression levels of both *OsLPR3* and *5* in the roots of –Pi (21 d) seedlings upon replenishment with + Pi (1 d). This provided evidence towards their transcriptional regulation by Pi availability and their potential roles in the maintenance of Pi homeostasis. Although there were significant increases in the relative expression levels of *OsLPR4* during long-term (7 d and 21 d) –Pi treatments compared with + Pi, subsequent replenishment with + Pi did not exert any attenuating effect on its elevated relative expression level. This suggested an unlikely role of Pi in the transcriptional regulation of *OsLPR4.* Overall, differential relative expression levels of *OsLPR1,3,4* and *5* during temporal –Pi treatments and after replenishment with + Pi suggested their specific roles in Pi sensing and signaling cascades. It is not surprising because members of a gene family often exhibit lack of functional redundancy. For instance, members of Pi transporter family (*OsPTs*) in rice exhibit variable responses to –Pi condition and play diverse roles in maintaining Pi homeostasis [44, 50–53].

### Split-root experiment revealed the effect of systemic Pi sensing on the relative expression levels of *OsLPR3*/*5*

**Fig. 8:**
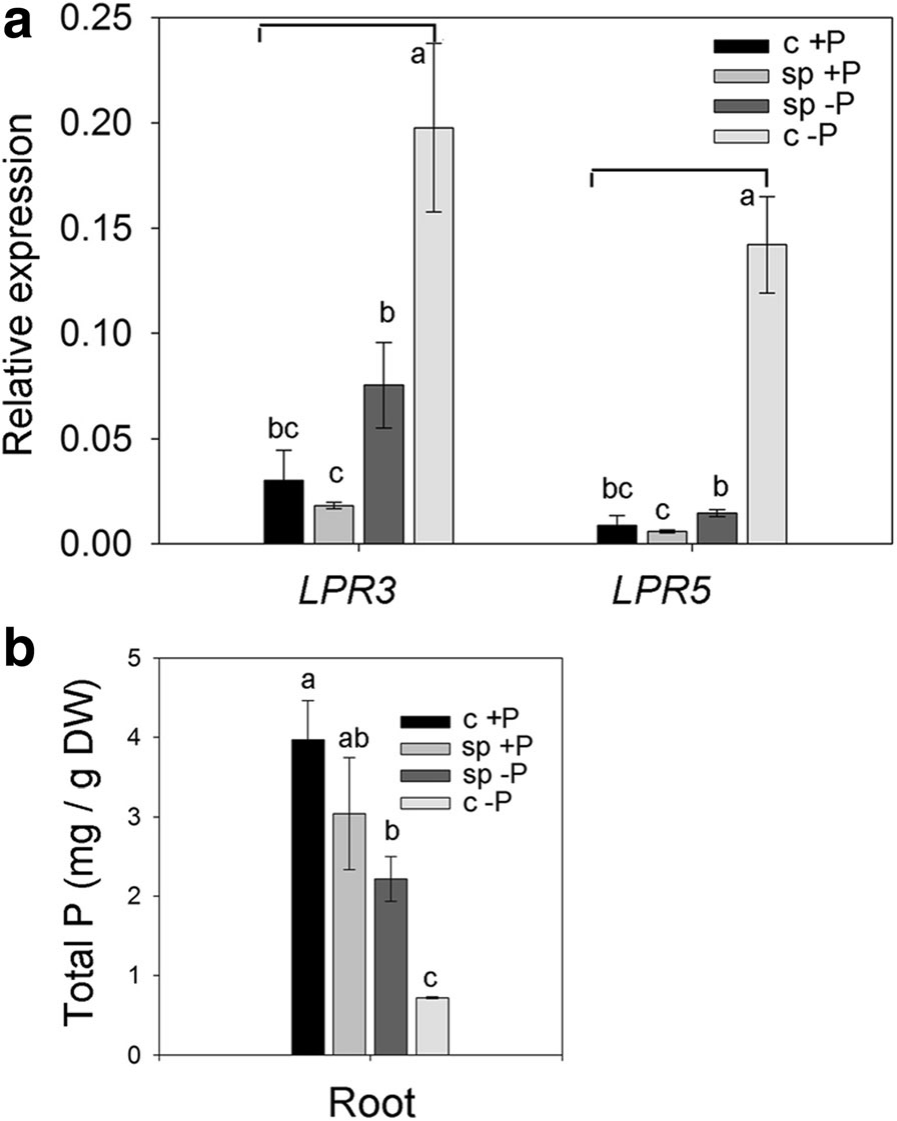
Relative expression of *OsLPR3/5* and total P concentration in split-root experiment. Intact roots of rice seedlings were divided into two halves with one half placed in 300 μM Pi (sp + P) and another half in 0 μM Pi (sp -P). As controls, both halves were grown under 300 μM Pi (c + P) and 0 μM Pi (c -P). **a** qRT-PCR was used for determining the relative expression levels of *OsLPR3/5* in the roots. *Actin* was used as an internal control. **b** Total P content. Values are means ± SE (*n* = 3) with different letters indicating values that differ significantly (*P* < 0.05)

Split-root experiment in which each half of the intact root system remains in contact with a different nutrient medium is an attractive technique for determining whether PSR genes are regulated by external Pi availability (local sensing) or by internal Pi status of the whole plant (systemic sensing) [54]. In Arabidopsis, using this technique, an array of PSR genes were identified that were specifically regulated either by local or systemic Pi sensing [55]. Therefore, in the present study, this technique was employed for determining the effects of local and systemic Pi sensing on the relative expression levels of *OsLPR3/5* and total P content in the root of rice seedlings (Fig. 8). In a hydroponic system, both halves of rice root were submerged either in + P (300 μM Pi) or –Pi (0 μM Pi) to mimic control plants growing in a homogeneous medium and hereafter referred as c + P and c –P, respectively. In another set-up, each half of the intact root system was placed in + P and –Pi nutrient media and referred to as sp + P and sp –P, respectively. qRT-PCR was employed for determining the relative expression levels of *OsLPR3/5* in the roots of the seedlings grown under c + P, c –P, sp + P and sp –P conditions (Fig. 8a). As anticipated, relative expression levels of *OsLPR3/5* were significantly higher in the roots of c –P compared with c + P. However, there were significant attenuations in their relative expression levels in sp –P roots compared with c –P and the values were almost comparable with c + P. Relative expression levels of *OsLPR3/5* were comparable in c + P and sp + P roots. This clearly suggested that despite the presence of sp –P roots in –Pi medium, the expression levels of *OsLPR3/5* were regulated systemically by whole plant Pi status. The results were contrary to an earlier study in Arabidopsis in which *LPR1* and *LPR2* were shown to play pivotal roles in local Pi sensing-mediated responses of PRG [6]. This suggested functional divergence of *LPR* family in taxonomically diverse Arabidopsis and rice. Root tissues were also analyzed for the total P content (Fig. 8b). Total P content was highest and lowest in c + P and c –P roots, respectively. Interestingly though, differences in the total P content in sp + P and sp –P were statistically insignificant. Variable total P content in these root tissues correlated with the *OsLPR3/5* expression levels in them.

### *OsLPR3/5* are negatively regulated by *OsPHR2* and are influenced by *SIZ1/PHO2/SPX1*-mediated Pi sensing

**Fig. 9:**
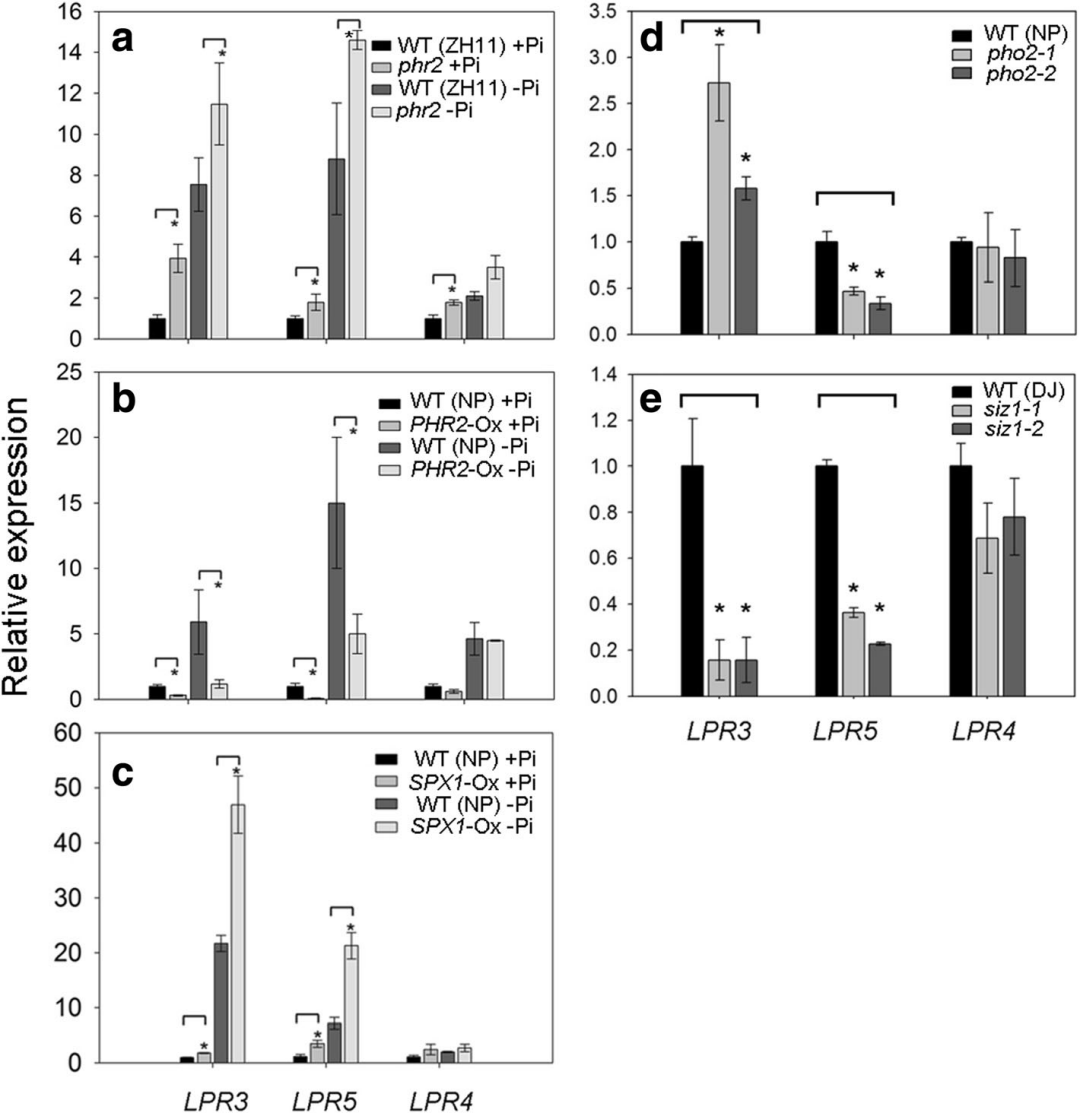
*OsLPRs* are differentially influenced by PHR2-mediated Pi sensing and signaling cascade. Seedlings (14-d-old) of **a**–**c** *osphr2*, plants overexpressing (Ox) *OsPHR2* and *OsSPX1* and their corresponding wildtypes (ZH11 and NP) were grown under + P (300 μM Pi) and–P (0 μM Pi) conditions and **d**, **e** *pho2-1*, *pho2-2*, *siz1-1* and *siz1-2* and their corresponding wildtypes (NP and DJ) under + P condition for 7 d. qRT-PCR was used for determining the relative expression levels of *OsLPR3/4/5 i*n the roots. *Actin* was used as an internal control. Values are means ± SE (*n* = 3) and asterisk indicates that the values of the mutants and Ox plants differ significantly (*P* < 0.05) compared with their corresponding wild types

In rice, several transcription factors (TFs) have been identified that play pivotal roles in the transcriptional regulation of PSR genes [3, 17, 56]. Among these TFs, *OsPHR2* is expressed constitutively under different Pi regime and has been implicated in regulating Pi signaling and homeostasis [18, 19]. To determine whether *OsPHR2* exerts any regulatory influence on *OsLPRs,* their relative expression levels were assayed in the roots of the wildtype (ZH11) and *osphr2* seedlings grown under + P and –P conditions (Fig. 9a). There were significant increases in the relative expression levels of *OsLPR3* and *5* in the roots of *osphr2* under both + P and –P conditions compared with their corresponding wild types. However, marginal but significant increase in the relative expression of *OsLPR4* was detected in the roots of *osphr2* compared with the wild type only under + P condition. Further, relative expression levels of *OsLPR3/4/5* were compared in + P and –P roots of *OsPHR2*-Ox plants and their corresponding wild types (Fig. 9b). Although relative expression levels of *OsLPR3* and *OsLPR5* were significantly attenuated in + P and –P roots of *OsPHR2*-Ox plants compared with their corresponding wild types, no such effect was detected in the relative expression levels of *OsLPR4*. This suggested a more pronounced negative regulatory influence of *OsPHR2* on the expression of *OsLPR3* and *5* than on *OsLPR4.* Interestingly though, the promoter of *OsLPR4* is enriched with P1BS motif*,* while those of other *OsLPRs* (*1*–*3*) are enriched with W-box motif (Additional file 7). In a global microarray analysis of Pi deficiency responses in Arabidopsis, promoters of the PSR genes were analyzed for the presence of P1BS motif(s) [31]. The analysis revealed enrichment of the promoters of several PSR genes with P1BS motif(s). In addition, several genes were also identified that were not induced under –P condition despite the presence of this motif. For instance, promoters of genes encoding purple acid phosphatase (*PAP*) *19* (At3g46120) and *20* (At3g52780) are enriched with 3 P1BS motifs each but neither of them shows any induction during Pi deficiency. In this context, it is not surprising to observe lack of any significant effect of either mutation (−P) or overexpression (+P and –P) of *OsPHR2* on the relative expression levels of *OsLPR4*. However, lack of P1BS motif(s) on the promoters of *OsLPR3/5* suggested their negative regulation by OsPHR2 by possibly invoking a feed-forward regulatory loop (FFRL). In Arabidopsis, TFs *NAM/ATAF1/2/CUC2016* (*NAC016*; At1g34180) and *NAC-LIKE, ACTIVATED BY AP3/PI* (*NAP*; At1g69490) repress the transcription of *ABSCISIC ACID-RESPONSIVE ELEMENT BINDING PROTEIN1* (*AREB1*; At1g45249) through a FFRL [57]. Presence of two W-box in the promoter of *OsLPR3* (Additional file 7) suggested a likely regulatory influence of WRKY TFs. In rice, WRKY TF superfamily comprises 109 members [58]. Recent study has shown the role of *OsWRKY74* in regulating Pi homeostasis [59]. Therefore, it would be intriguing to investigate whether *OsWRKY74* and OsPHR2 regulate *OsLPR3* in a FFRL, which warrants further studies.

Transcript levels of *OsSPX1* induced in –P root and stem and also in *OsPHR2*-Ox plants suggesting the former to be downstream of the latter [60]. Another study demonstrated the inhibition in the activity of OsPHR2 by OsSPX1 in a Pi-dependent manner [22]. Together these studies suggested a negative feedback loop regulation of *OsPHR2* by *OsSPX1.* Since the relative expression levels of *OsLPR3* and *OsLPR5* were significantly increased in *osphr2* under both + P and –P conditions (Fig. 9a), a similar expression pattern was anticipated in *SPX1*-Ox. Consistent with this assumption, significant increases in the relative expression levels of *OsLPR3* and *OsLPR5* were observed in *SPX1*-Ox under both + P and –P conditions compared with their corresponding wild types (Fig. 9c). On the contrary, relative expression levels of *OsLPR4* in *SPX1*-Ox (+P and –P) were comparable with the wild type. This suggested that *OsLPR3* and *OsLPR5* are part of *OsPHR2*-*OsSPX1*-mediated regulation of Pi homeostasis.

*OsPHO*2, a signaling component downstream of *OsPHR2*, plays a key role in regulating the expression of *OsPTs* and multiple Pi starvation responses thereby influencing Pi utilization in rice [20, 21]. Therefore, the regulatory influence of *OsPHO2* on *OsLPR3-5* was investigated (Fig. 9d). There were significant increases in the relative expression levels of *OsLPR3* in *pho2-1* and *pho2-2* compared with the wild type. An increased expression of *OsSPX1* in the roots of *pho2* mutant suggested a negative regulatory influence of *OsPHO2* on its downstream *OsSPX1* [60]. The accentuated relative expression levels of *OsLPR3* in *SPX1*-Ox (Fig. 9c) and *pho2-1* and *pho2-2* (Fig. 9d) thus suggested it to be downstream of *OsPHR2-OsPHO2-OsSPX1* pathway. On the contrary, significant reductions and no effect on the relative expression levels of *OsLPR5* and *OsLPR4,* respectively in *pho2-1* and *pho2-2* compared with the wild type (Fig. 9d) highlighted differential roles of the members of *OsLPR* family in *OsPHR2-OsPHO2-OsSPX1-*mediated Pi sensing.

Sumoylation is a critical post-translational modification involved in protein-protein interaction, transcriptional activation and localization of proteins [61]. *OsSIZ1* and *OsSIZ2*, homologs of Arabidopsis *SIZ1* in rice, partially complemented the morphological phenotype of *siz1-2* in Arabidopsis [62]. Further, several genes involved in Pi sensing and signaling were modulated in *ossiz1* [63]. Therefore, the effects of *OsSIZ1* on the regulation of *OsLPR3-5*, were assayed (Fig. 9e). Significant reductions were observed in the relative expression levels of *OsLPR3* and *OsLPR5* in both *siz1-1* and *siz1-2* compared with the wild-type. Although marginal reductions in the expression levels of *OsLPR4* were also detected in these mutants, the values were statistically insignificant. This suggested a post-translational regulatory influence of *OsSIZ1* on *OsLPR3* and *OsLPR5.* However, at present it is not known whether *OsSIZ1* exerts direct regulatory influence on *OsLPR3* and *OsLPR5* by sumoylating them or mediated through a target, which is yet to be identified. Functional characterization of *OsLPRs* could provide an insight into their specific roles in maintaining Pi homeostasis and thus warrants further studies.

## Conclusion

This study presented a detailed genome-wide analysis of the gene structure, phylogenetic evolution and tissue-specific expression patterns of LPR family members in rice (*OsLPR1- OsLPR5*). Phylogenetic analysis revealed their grouping into two distinct subclades. Differential expression of these genes under deficiencies of Pi and other nutrients suggested lack of functional redundancy across them. Further an insight into the likely roles of *OsLPR3* and *OsLPR5* in the maintenance of Pi homeostasis was gained by assaying their relative expression levels in loss-of-function mutants (*ossiz1, osphr2* and *ospho2*) and transgenic rice overexpressing either *OsPHR2* or *OsSPX1*. The results from this study thus provide a basis for further detailed functional characterization of different members of *OsLPR* family for elucidation of their specific roles in maintaining homeostasis during deficiency of Pi and/or other nutrients.

## Methods

### Database searches, sequence alignment and phylogenetic analysis

Complete genomic sequence and transcripts of *OsLPR1-5* were retrieved from Michigan State University (MSU) Rice Genome Annotation Project assembly (v7) (http://rice.plantbiology.msu.edu/). Identification of *LPR* homologs was performed using tBLASTn program and PLAZA1.0 database (http://bioinformatics.psb.ugent.be/plaza/). *LPR* homologs were identified in dicots (*Arabidopsis thaliana*, *Capsella rubella, Carica papaya, Cicer arietinum, Cucumis sativus, Fragaria vesca*, *Glycine max*, *Lotus japonicus, Malus domestica*, *Manihot esculenta*, *Populus trichocarpa*, *Prunus persica*, *Ricinus communis*, *Solanum lycopersicum*, *Theobroma cacao* and *Vitis vinifera*), monocots (*Aegilop stauschii, Brachypodium distachyon, Hordeum vulgare*, *Oryza sativa, Setaria italica*, *Sorghum bicolor, Triticum urartu* and *Zea mays*), gymnosperms (*Picea sitchensis* and *Selaginella moellendorffii*), bryophytes (*Physcomitrella patens*) and chlorophyta (*Volvox carteri* and *Chlamydomonas reinhardtii*). The unrooted phylogenetic tree of *LPR* homologs was made using the neighbor-joining method and displayed using the MEGA4.0 program.

### Plant materials and growth conditions

In the present study, wild type rice (*Oryza sativa*) ssp. *japonica* varieties (Nipponbare, ZH11 and Dongjin), T-DNA insertion mutants (*ospho2-1/2* [21], *ossiz1-1/2* [63], *osphr2* [64] in the backgrounds of Nipponbare, Dongjin and ZH11, respectively) and two homozygous overexpresors (*OsSPX1-*Ox [60] and *OsPHR2-*Ox [Gu unpublished work] in Nipponbare background) were used. For *OsPHR2* overexpressors, the ORF of *OsPHR2* was amplified using the specific primers from Nipponbare cDNA. The PCR product was ligated into the pTCK303 vector as described [44]. By electroporation, the construct was transferred to *Agrobacterium tumefaciens* strain EHA105 and then transformed into Nipponbare as described [65]. For hydroponic experiments, rice seeds were surface-sterilized for 1 min with 75 % ethanol (v/v) and for 30 min with diluted (1:3, v/v) NaClO followed by thorough rinsing for 30 min with deionized water. Seeds were germinated in dark at 25 °C for 3 d. The hydroponic experiments were carried out in a growth room with a 16-h-light (30 °C)/8-h-dark (22 °C) photoperiod and the relative humidity was maintained at approximately 70 %. Uniformly grown seedlings (7-d-old) were then transferred to complete nutrient solution containing 1.25 mM NH_4_NO_3_, 300 μM KH_2_PO_4_, 0.35 mM K_2_SO_4_, 1 mM CaCl_2_ · 2H_2_O, 1 mM MgSO_4_ · 7H_2_O, 0.5 mM Na_2_SiO_3_ · 9H_2_O, 20 μM Fe-EDTA, 20 μM H_3_BO_3_, 9 μM MnCl_2_ · 4H_2_O, 0.32 μM CuSO_4_ · 5H_2_O, 0.77 μM ZnSO_4_ · 7H_2_O and 0.39 μM Na_2_MoO_4_ · 2H_2_O. For + P (control) and –P media, KH_2_PO_4_ concentrations used were 300 μM and 0 μM, respectively. To maintain equimolar concentration of K in + P and –P media, KH_2_PO_4_ in + P medium was replaced with K_2_SO_4_ in –P medium. For + K (control) and –K media, 300 μM KH_2_PO_4_ and 300 μM NaH_2_PO_4_ were used, respectively. For + Mg (control) and –Mg media, 1 mM MgSO_4_ · 7H_2_O and 1 mM Na_2_SO_4_ · 7H_2_O were used, respectively. For –Fe medium, 20 μM Fe-EDTA was eliminated from + Fe (control) medium. Deionized water was used throughout the experiments and pH of all the nutrient solutions were adjusted to 5.0. For all the experiments, nutrient solutions in the hydroponic set up were refreshed every 3rd d. For Pi split-root experiment, seedlings were prepared and grown in complete nutrient solution for 14 d, and then transferred to split-root container for 14 d. The roots of individual plants were separated into two equal parts, placed into separate containers such that one half received 300 μM Pi, while the other half did not receive any Pi. The controls included a split-root treatment in which both halves of the roots received + Pi (300 μM Pi) and –P (0 μM Pi). For Phi treatment, seedlings were grown in –Pi (0 μM Pi) for 21 d. Uniformly grown seedlings were then transferred to + Pi (300 μM Pi), −Pi (0 μM Pi) and + Phi/–Pi (300 μM Phi, 0 μM Pi) solutions for 3 d.

### qRT-PCR

Total RNAs from various tissues were isolated using Trizol reagent (Invitrogen) and first-strand cDNA was synthesized with an oligo (dT)-18 primer and reverse transcriptase. *OsActin* (accession no. AB047313) was used as an internal control for qRT-PCR analysis. qRT-PCR analysis was performed using SYBR green master mix (Vazyme) and ABI StepOnePlus Sequence Detection System (Applied Biosystems), from biological triplicates. Primers used for qRT-PCR are listed in Additional file 8.

### Measurements of Pi and total P concentrations in plants

To measure Pi concentration in plants, about 0.5 g Fresh sample was used for the quantification of Pi concentration in plants as described [18]. Total P concentration was quantified by digesting dry sample (0.05 g) with H_2_SO_4_-H_2_O_2_ at 280 °C followed by assay with molybdenum blue as described [66].

### Statistical analysis

Data were analyzed by analysis of variance (ANOVA) using the SPSS 13 program. Different letters or asterisks on the histograms between the mutants and the WT and/or different treatments indicate their statistically significant difference using Duncan multiple range test at *P* < 0.05.

## Abbreviations

AREB1, *ABSCISIC ACID-RESPONSIVE ELEMENT BINDING PROTEIN*1; Fe, iron; FFRL, feed-forward regulatory loop; K, Potassium; LTN1, *LEAF TIP NECROSIS*1; LPR1, low phosphate root1; lpsi, local phosphate sensing impaired; Mg, magnesium; MCOs, multicopper oxidases; MSU, Michigan State University; N, nitrogen; NAC016, NAM/ATAF1/2/CUC2016; NAP, *NAC-LIKE, ACTIVATED BY AP3/PI*; NCBI, National Center for Biotechnology Information; Ox, overexpressing; P, phosphorus; Pi, phosphate; Phi, phosphite; −P, Pi deficiency; P1BS, PHR1-binding sequence; PSR, Pi starvation-responsive; PRG, primary root growth; PAP, purple acid phosphatase; SUMO, small ubiquitin-like modifier; qRT-PCR, quantitative real-time PCR; QTLs, quantitative trait loci; SI, sequence identity; TF, transcription factor; TNC, trinuclear Cu cluster; UTR, untranslated region

## Additional files

Additional file 1: Details of locus ID, cDNA accession number and protein characteristics of the members of *OsLPR* gene family. (DOC 33 kb) Additional file 2: Schematic figure showing positions of *OsLPR1-5* on rice chromosome1. (DOC 84 kb) Additional file 3: Analysis of domain structure of *LPRs* in diverse plant species. (DOC 101 kb) Additional file 4: Phylogenetic analysis of the members of MCO family in rice. (DOC 288 kb) Additional file 5: Alignment of amino acid sequences of LPR proteins in rice and Arabidopsis. (DOC 390 kb) Additional file 6:
*OsLPRs* exhibited tissue-specific expression. (DOC 75 kb) Additional file 7:
*Cis*-elements in the promoters of *OsLPRs. (DOC 78 kb)*
Additional file 8: Primers used for qRT-PCR analysis of *OsLPR*sand *OsPT6*. (DOC 32 kb)
