# Supplementary material for: Identification and expression analysis of OsLPR family revealed the potential roles of OsLPR3 and 5 in maintaining phosphate homeostasis in rice
Source: BMC Plant Biol. 2016 Oct 3;16:210. doi: 10.1186/s12870-016-0853-x (PMC5048653; doi:10.1186/s12870-016-0853-x)
Supplement: Additional file 1: — Details of locus ID, cDNA accession number and protein characteristics of the members of OsLPR gene family. (DOC 33 kb) [file 12870_2016_853_MOESM1_ESM.doc]

|  | Rice locus | |  | Protein | | |
| --- | --- | --- | --- | --- | --- | --- |
| Gene | MSU | RAP_DB | cDNA accession number | Number of amino acids | MW (kDa) | *p*l |
| *OsLPR1* | LOC_Os01g03530 | Os01g0126100 | AK106990 | 583 | 64.1 | 6.94 |
| *OsLPR2* | LOC_Os01g03549 | Os01g0126200 | AK121464 | 599 | 65.8 | 7.22 |
| *OsLPR3* | LOC_Os01g03630 | Os01g0127000 | AK105526 | 535 | 59.6 | 5.73 |
| *OsLPR4* | LOC_Os01g03620 | Os01g0126900 | AK109876 | 596 | 65.2 | 6.51 |
| *OsLPR5* | LOC_Os01g03640 | Os01g0127200 | AK243237 | 638 | 70.9 | 7.1 |

**Additional file 1: Details of locus ID, cDNA accession number and protein** characteristics **of the members of *LPR* family in rice**
