# Supplementary material for: Identification and expression analysis of OsLPR family revealed the potential roles of OsLPR3 and 5 in maintaining phosphate homeostasis in rice
Source: BMC Plant Biol. 2016 Oct 3;16:210. doi: 10.1186/s12870-016-0853-x (PMC5048653; doi:10.1186/s12870-016-0853-x)
Supplement: Additional file 2: — Schematic figure showing positions of OsLPR1-5 on rice chromosome1. (DOC 84 kb) [file 12870_2016_853_MOESM2_ESM.doc]

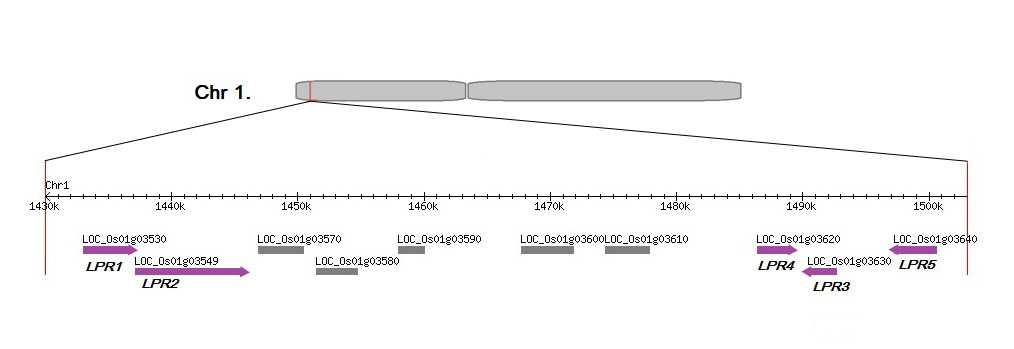


**Additional file 2: Schematic figure showing positions of *LPR* genes on rice chromosome 1.** Purple arrows indicate their lengths and directions of transcription.
