# Supplementary material for: Identification and expression analysis of OsLPR family revealed the potential roles of OsLPR3 and 5 in maintaining phosphate homeostasis in rice
Source: BMC Plant Biol. 2016 Oct 3;16:210. doi: 10.1186/s12870-016-0853-x (PMC5048653; doi:10.1186/s12870-016-0853-x)
Supplement: Additional file 3: — Analysis of domain structure of LPRs in diverse plant species. (DOC 101 kb) [file 12870_2016_853_MOESM3_ESM.doc]

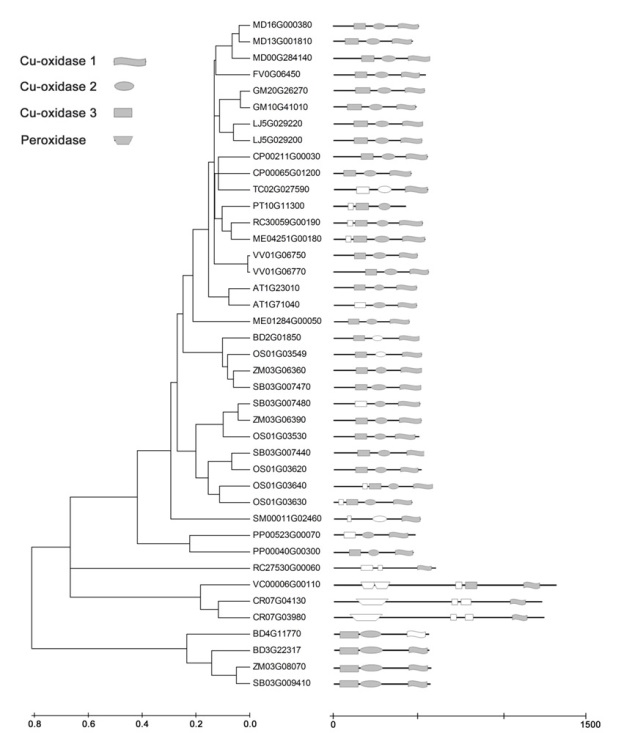


**Additional file 3: Analysis of domain structure of *LPRs* in diverse plant species.** Pfam and NCBI Protein Database were used for predicting the domain structure of OsLPR proteins. Cu-oxidase I, II, III and peroxidase domains are indicated by streamer, elliptical, rectangular and trapezoidal symbol, respectively. Solid and empty symbol represents significant and insignificant domains, respectively.
