# Supplementary material for: Identification and expression analysis of OsLPR family revealed the potential roles of OsLPR3 and 5 in maintaining phosphate homeostasis in rice
Source: BMC Plant Biol. 2016 Oct 3;16:210. doi: 10.1186/s12870-016-0853-x (PMC5048653; doi:10.1186/s12870-016-0853-x)
Supplement: Additional file 4: — Phylogenetic analysis of the members of MCO family in rice. (DOC 288 kb) [file 12870_2016_853_MOESM4_ESM.doc]

**
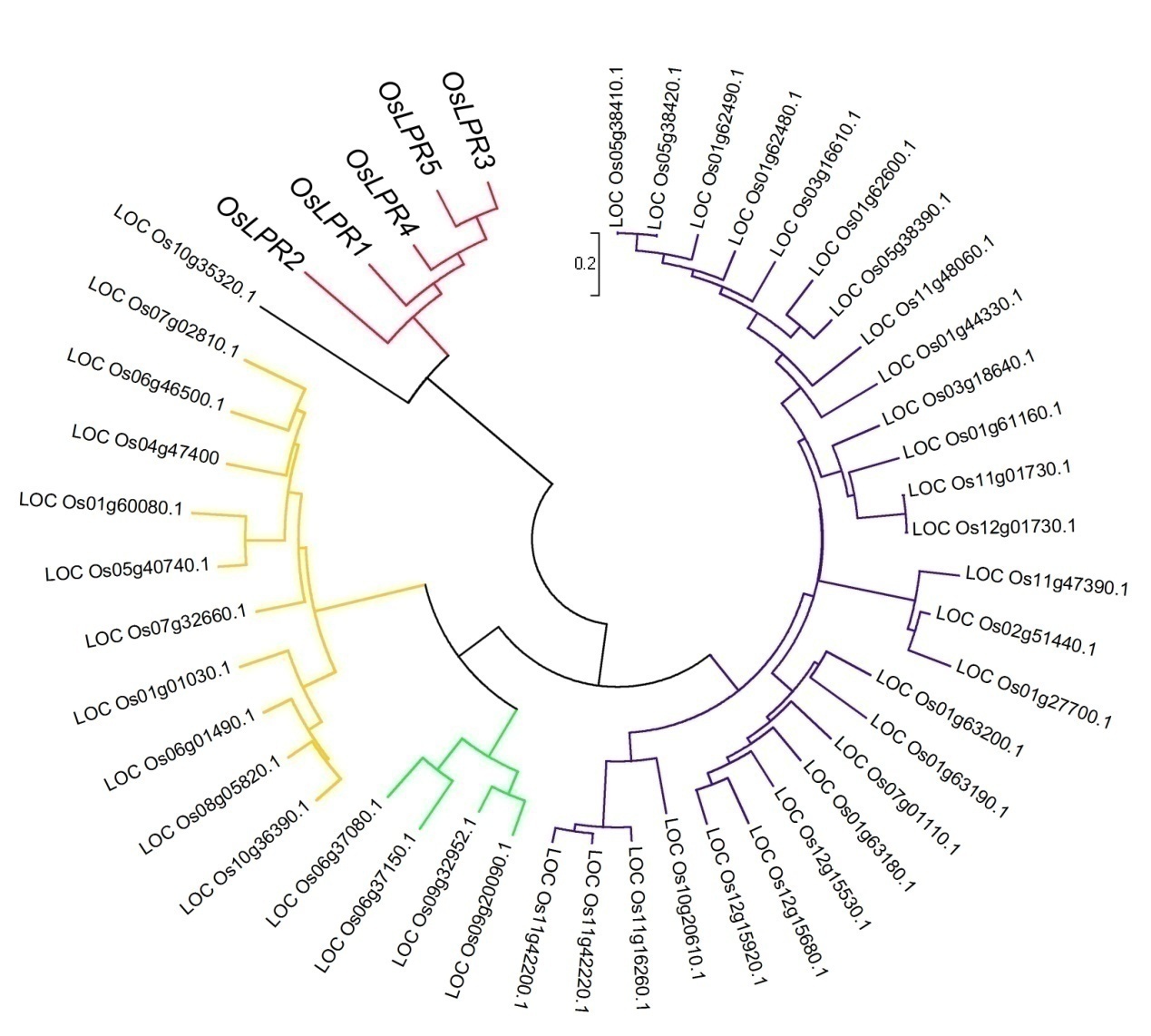
**

**Additional file 4: Phylogenetic analysis of the members of MCO family in rice.** Unrooted dendrogram was derived by aligning protein sequences of OsLPRs (red branch), laccases (blue branch), L-ascorbate oxidases (green branch), mono- copper oxidases (yellow branch) and LOCOs10g35320.1 (black branch).
