# Supplementary material for: Identification and expression analysis of OsLPR family revealed the potential roles of OsLPR3 and 5 in maintaining phosphate homeostasis in rice
Source: BMC Plant Biol. 2016 Oct 3;16:210. doi: 10.1186/s12870-016-0853-x (PMC5048653; doi:10.1186/s12870-016-0853-x)
Supplement: Additional file 5: — Alignment of amino acid sequences of LPR proteins in rice and Arabidopsis. (DOC 390 kb) [file 12870_2016_853_MOESM5_ESM.doc]

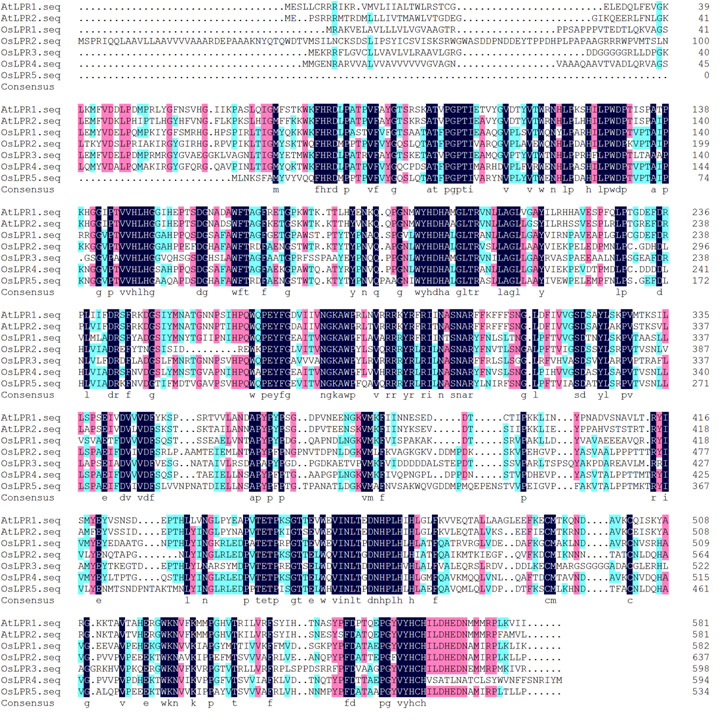


**Additional file 5: Alignment of amino acid sequences of LPR proteins.** ClustalX program was used for determining identical and conversed amino acid residues indicated in navy blue and pink background, respectively across LPR proteins in rice and Arabidopsis**.**
