# Supplementary material for: Identification and expression analysis of OsLPR family revealed the potential roles of OsLPR3 and 5 in maintaining phosphate homeostasis in rice
Source: BMC Plant Biol. 2016 Oct 3;16:210. doi: 10.1186/s12870-016-0853-x (PMC5048653; doi:10.1186/s12870-016-0853-x)
Supplement: Additional file 6: — OsLPRs exhibited tissue-specific expression. (DOC 75 kb) [file 12870_2016_853_MOESM6_ESM.doc]

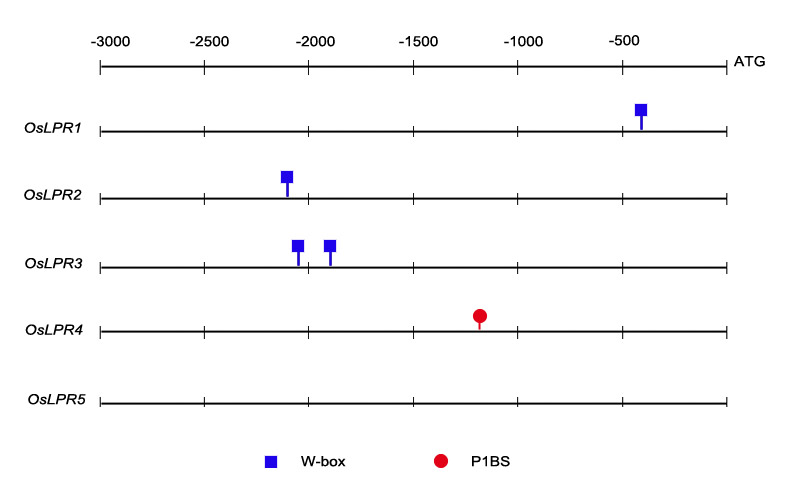


**Additional file 6: Pi-regulated *cis*-elements in the promoters of *OsLPRs.*** W-box (TTGACY) and P1BS (GNATATNC) motifs are represented with blue square and red circle, respectively.
