# Supplementary material for: Identification and expression analysis of OsLPR family revealed the potential roles of OsLPR3 and 5 in maintaining phosphate homeostasis in rice
Source: BMC Plant Biol. 2016 Oct 3;16:210. doi: 10.1186/s12870-016-0853-x (PMC5048653; doi:10.1186/s12870-016-0853-x)
Supplement: Additional file 7: — Cis-elements in the promoters of OsLPRs. (DOC 78 kb) [file 12870_2016_853_MOESM7_ESM.doc]

***
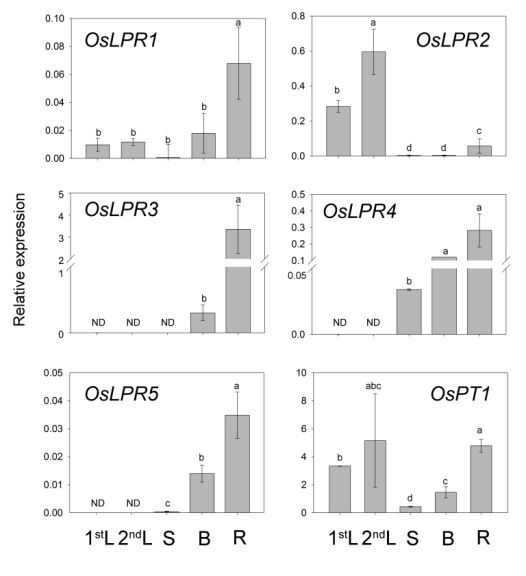
***

**Additional file 7： *OsLPRs* revealed differential tissue-specific expression.** Tissues were collected at seedling (21-d-old) stage. Leaf blades were named as 1st to 4th from top to base. qRT-PCR was used for determining the relative expression levels of *OsLPRs* in 1st leaf blade (1^st^ L), 4st leaf blade (2^nd^ L), leaf sheath (S), basal stem (B) and root (R) of 21d rice seedling. *OsPT1* (Os01g0630200) was used as a positive control. Actin (*OsRac1*; accession no. AB047313) was used as an internal control. Values are means ± SE (*n* = 3) and different letters indicates that the values differ significantly (P < 0.05).
