# Supplementary material for: Identification and expression analysis of OsLPR family revealed the potential roles of OsLPR3 and 5 in maintaining phosphate homeostasis in rice
Source: BMC Plant Biol. 2016 Oct 3;16:210. doi: 10.1186/s12870-016-0853-x (PMC5048653; doi:10.1186/s12870-016-0853-x)
Supplement: Additional file 8: — Primers used for qRT-PCR analysis of OsLPRsand OsPT6. (DOC 32 kb) [file 12870_2016_853_MOESM8_ESM.doc]

**Additional file 8: Primers used for qRT-PCR analysis of *OsLPR*s** , ***OsPT6* and *OsPT1.***

| **Gene name** | **Forward primer (5′-3′)** | **Reverse primer (5′-3′)** |
| --- | --- | --- |
| *OsActin* | GGGTTCACAAGTCTGCCTATTGT | ACGGGACACGACCAAGGA |
| *OsLPR1* | AAGCTGTAGGCCATGTGTTTGTAC | CATAGTTTTTTGCTTTCTGTCTCCTAAA |
| *OsLPR2* | CCACGTCCGTGTGCAGTTC | TCACGTGGGTAGATACACATATATAGGA |
| *OsLPR3* | TGCATGGTTGTCTCCATTCG | CAAGGCAAACGTACAACAAAGG |
| *OsLPR4* | CCTCTCCAGCTGCTTCATCTG | GCTCGATTCTCACCCATCATC |
| *OsLPR5* | CGATGAGAATATGAGATGAAGAAGCT | CGCACCAGTTTATGACTAGCAAA |
| *OsPT6* | CCGCCCCTGCAAACTGTA | GAACTGGCGGTTTCTTCGAT |
| *OsPT1* | CGCTTCCGTACGAGTGGTAGT | GGTTCTTTCAAATCCAGGGAAA |
